# Case report: cognitive performance in an extreme case of anorexia nervosa with a body mass index of 7.7

**DOI:** 10.1186/s12888-020-02701-1

**Published:** 2020-06-05

**Authors:** Simone Daugaard Hemmingsen, Mia Beck Lichtenstein, Alia Arif Hussain, Jan Magnus Sjögren, René Klinkby Støving

**Affiliations:** 1 Centre for Eating Disorder, Odense University Hospital, Odense, Denmark; 2 Research Unit for Medical Endocrinology, Odense University Hospital, Odense, Denmark; 3 Department of Clinical Research, University of Southern Denmark, Odense, Denmark; 4 Open Patient data Explorative Network (OPEN), Odense, Denmark; 5 The Research Unit, Child and Adolescent Psychiatry, Mental Health Services in the Region of Southern Denmark, Odense, Denmark; 6 Centre for Telepsychiatry, Mental Health Services in the Region of Southern Denmark, Odense, Denmark; 7 Department of Psychology, University of Southern Denmark, Odense, Denmark; 8 Eating Disorder Unit, Mental Health Centre Ballerup, Mental Health Services in the Capital Region of Denmark, Copenhagen, Denmark; 9 Department of Clinical Medicine, University of Copenhagen, Copenhagen, Denmark

**Keywords:** Anorexia nervosa, Cognitive performance, Neuropsychology, Emaciation, Undernutrition

## Abstract

**Background:**

Studies show that adult patients with anorexia nervosa display cognitive impairments. These impairments may be caused by illness-related circumstances such as low weight. However, the question is whether there is a cognitive adaptation to enduring undernutrition in anorexia nervosa. To our knowledge, cognitive performance has not been assessed previously in a patient with anorexia nervosa with a body mass index as low as 7.7 kg/m2.

**Case presentation:**

We present the cognitive profile of a 35-year-old woman with severe and enduring anorexia nervosa who was diagnosed at the age of 10 years. She was assessed with a broad neuropsychological test battery three times during a year. Her body mass index was 8.4, 9.3, and 7.7 kg/m^2^, respectively. Her general memory performance was above the normal range and she performed well on verbal and design fluency tasks. Her working memory and processing speed were within the normal range. However, her results on cognitive flexibility tasks (set-shifting) were below the normal range.

**Conclusions:**

The case study suggests that it is possible to perform normally cognitively despite extreme and chronic malnutrition though set-shifting ability may be affected. This opens for discussion whether patients with anorexia nervosa can maintain neuropsychological performance in spite of extreme underweight and starvation.

**Trial registration:**

ClinicalTrials.gov, NCT02502617. Registered 20 July 2015.

## Background

A growing amount of evidence indicate that anorexia nervosa (AN) is associated with impaired or inefficient neuropsychological performance in relation to healthy control subjects, regarding attention [1, 2], memory [1–4], processing speed [4], and especially the executive functions [5] central coherence [6], decision-making [6, 7], and cognitive flexibility [8, 9]. It has been debated whether this is related to state (due to factors such as malnutrition) or trait (a premorbid trait or endophenotype of the disorder [10]). Some studies have found that patients who recovered from AN have impaired cognitive performance compared to healthy control subjects [11, 12], supporting the trait theory of the disorder. However, longitudinal studies have found that executive functions can be normalized following weight stabilization in patients with AN [13, 14], supporting the state theory.

Research on cognitive performance before and after re-nutrition in adult patients with extreme and chronic AN is sparse. Some studies have examined cognitive performance in patients with AN with a mean body mass index (BMI) below 15 kg/m2 (e.g. [10]), corresponding to extreme AN severity according to the Diagnostic and Statistical Manual of Mental Disorders 5 (DSM-5) [15]. However, it is unclear if patients with AN with BMI below 10 kg/m2 will display the same cognitive profile.

It has been suggested that malnutrition might affect cognitive performance since the classic Minnesota Semi-Starvation Experiment [16], where cognitive functions were studied in 36 healthy military objectors with normal weight before and after semistarvation with 25% weight-loss over a 24-week period. The men reported decline in concentration. However, the standardized tests that were administered did not confirm measurable alterations. Newer research on healthy subjects, although somewhat inconclusive, indicates affected psychomotor speed and executive functions following short-term semi-starvation [17].

However, other factors than malnutrition or weight-loss have been suggested to affect cognitive performance in patients with AN, such as long illness duration [18] and age [18]. This could explain a difference in results for children/adolescents and adults with AN mentioned in the literature [19, 20], which cannot be explained by the trait theory.

The current case report was part of an ongoing longitudinal research project investigating the effect of re-nutrition on cognitive performance in patients with severe AN. The aim of the case study was to present the neuropsychological performance of a patient with chronic AN and extremely low BMI in order to discuss whether extremely low weight and long duration of illness are associated with cognitive impairment and if cognitive adaptation takes place. No study to our knowledge has previously reported on the cognitive profile of a patient with AN with a BMI as low as 7.7 kg/m2.

We want to introduce the idea of cognitive adaptation to severe malnutrition as a supplement to the discussion on cognitive impairment in AN. However, this idea should not be confused with Taylor’s Theory of Cognitive Adaptation [21]. The presented idea of cognitive adaptation is the idea that cognitive functions can adapt to persisting low weight in AN, i.e. cognitive performance can remain normal or regain normality in severe and enduring AN. The adaptation does not exclude specific cognitive impairment.

## Case presentation

The current case report investigates the cognitive profile of a 35-year-old Caucasian woman with extremely severe and enduring AN who was diagnosed at the age of 10 years. The patient’s weight loss is accomplished through fasting. According to the DSM-5 [15], the patient’s symptoms are in accordance with the restricting type and the severity of AN for the patient is categorized as extreme. The patient has had low body weight since the onset of the disease 25 years ago. Consequently, she is still prepubescent.

The patient’s extreme malnutrition, the medical complications, and the refeeding treatment has previously been described in a case report [22]. Since the previous report [22], she has survived another 5 years, living in her own residence with several stabilizing hospitalizations. Her nadir BMI, defined as the lowest registered BMI, has decreased further to 7.2 kg/m2. To our knowledge, this is the lowest BMI reported in AN in the literature. During her long and severe illness course, she has participated in psychotherapy for years. However, during the past few years, she has refused to participate in psychotherapy, while she has continued the harm-reducing treatment in the nutrition department. No cognitive profile has been assessed before the current report.

She has continuously been provided supplementation with vitamins and minerals. At the present admission, she weighed 20.2 kg, including edema corresponding to at least 2 kg, and her height was 1.55 m, corresponding to a BMI of 8.41 kg/m^2^. After life-saving and stabilizing fluid and electrolyte correction, and refeeding according to guidelines [23] during 2 weeks of hospitalization, we tested her with a neuropsychological test battery (2 weeks after admission: T_0_). After an additional 2 months of hospitalization, she could not be motivated to continue the treatment any longer. Due to years of history with rapid relapse after prolonged forced treatment, she was allowed to be discharged to outpatient follow-up. She was re-tested in the outpatient clinic 6 days following dropout from inpatient treatment and approximately 3 months after admission, (re-test: T_1_) with a weight of 22.4 kg (BMI: 9.3 kg/m^2^), and again at 12 months from T_0_, during a re-hospitalization, 7 days after admission (follow-up: T_2_), with BMI 7.7 kg/m^2^. Thus, T_0_ and T_2_ were done at the hospital after initial stabilizing glycemic, fluid- and electrolyte correction, whereas T_1_ was done in an outpatient setting, where she was in a clinically stable condition, but without the initial stabilizing treatment.

### The psychopathological profile of the patient

The patient scored 21 on the Beck Depression Inventory II (BDI-II [24];) indicating moderate depression at 2 weeks after admission (T_0_). Her scores on the Eating Disorder Inventory 3 (EDI-3 [25];) at T_0_ are presented in Table 1 below. Compared to the Danish validation of EDI-3 for patients with AN ( [26]; Table 1), her low scores on the Drive for Thinness, the Interoceptive Deficits, the Perfectionism, and the Asceticism subscales are of interest.

**Table 1:** Eating Disorder Inventory 3 (EDI-3) scores for the patient and from the Danish validation of EDI-3^a^ on a sample of patients with anorexia nervosa (AN)

EDI-3 subscales	Scores in the present case of AN	Danish validation sample of AN (***N*** = 84)^**a**^***Mean (SD)***
Drive for thinness (DT)	7	18.7 (6.3)
Bulimia (B)	0	8.0 (8.0)
Body dissatisfaction (BD)	18	24.4 (9.3)
Low self-esteem (LSE)	21	13.8 (5.6)
Personal alienation (PA)	10	13.1 (5.3)
Interpersonal insecurity (II)	10	10.0 (5.6)
Interpersonal alienation (IA)	5	9.3 (5.4)
Interoceptive deficits (ID)	2	19.9 (7.6)
Emotional dysregulation (ED)	3	8.9 (5.1)
Perfectionism (P)	1	11.0 (4.6)
Asceticism (AS)	1	12.9 (6.0)
Maturity fear (MF)	9	12.2 (6.9)

^a^Clausen et al. [26]

### Qualitative observations

During the first 2 weeks after admission, the patient was unable to participate in the neuropsychological assessment due to fatigue. Two weeks after admission, when the baseline assessment took place (T_0_), the patient was lying down during the assessment and was noticeably tired. This was neither the case at retest (T_1_) nor at follow-up (T_2_) where the patient was sitting at a table. Her alertness and energy level at follow-up (T_2_) were notable in light of her low BMI. The patient was calm during all three assessments (divided into six sessions) and expressed that the tests were fun. The aim of the study was explained to the patient before the first administration. However, only information written in the test manuals was given during each assessment.

### Measures

The following validated neuropsychological tests were selected in cooperation with an experienced neuropsychologist to examine a wide range of cognitive functions: the Wechsler Memory Scale III (WMS-III) [27]; the d2-R Test of Attention – Revised [28, 29]; the Processing Speed Index (PSI) of the Wechsler Adult Intelligence Scale IV (WAIS-IV) [30]; the Delis-Kaplan Executive Function System (D-KEFS) [31], Verbal Fluency Test, Design Fluency Test and Trail Making Test; and the Wisconsin Card Sorting Test Revised and Expanded (WCST) [32] (only administered at T_0_). Information on each test variable, including internal consistency and test-retest reliability, are presented in Table 2. The test battery can be administered in approximately 2 h. For all three administrations, the test battery was divided into two sessions (1 h per session) 1 day apart.

**Table 2:** The neuropsychological test battery

Instrument	Variables	Description	Higher/lower scores indicating improvement	Reliability
Wechsler Memory Scale III (WMS-III)^1^	Logical memory I	Task: History recitation	Higher	Adequate internal consistency: 0.88^a^ Adequate test – retest: 0.77^b^ SE_M_: 1.04^a^ S_diff_: 1.47
	Logical Memory II	Task: Delayed history recitation	Higher	Low internal consistency: 0.84^a^ Adequate test – retest: 0.77^b^ SE_M_: 1.20^a^ S_diff_: 1.70
	Verbal Paired Associates I	Task: Word pairing	Higher	Adequate internal consistency: 0.95^a^ Adequate test – retest: 0.77^b^ SE_M_: 0.67^a^ S_diff_: 0.95
	Verbal Paired Associates II	Task: Delayed word pairing	Higher	Adequate internal consistency: 0.88^a^ Adequate test – retest: 0.73^b^ SE_M_: 1.04^a^ S_diff_: 1.47
	Faces I	Task: Face recognition among other faces	Higher	Low internal consistency: 0.77^a^ Low test – retest: 0.64^b^ SE_M_: 1.44^a^ S_diff_: 2.04
	Faces II	Task: Delayed face recognition	Higher	Low internal consistency: 0.83^a^ Low test – retest: 0.58^b^ SE_M_: 1.24^a^ S_diff_: 1.75
	Family Pictures I	Task: Picture recall	Higher	Adequate internal consistency: 0.86^a^ Low test – retest: 0.61^b^ SE_M_: 1.12^a^ S_diff_: 1.58
	Family Pictures II	Task: Delayed picture recall	Higher	Adequate internal consistency: 0.85^a^ Low test – retest: 0.67^b^ SE_M_: 1.16^a^ S_diff_: 1.64
	Letter-Number Sequencing	Task: Recall and ordering letters and numbers	Higher	Low internal consistency: 0.75^a^ Low test – retest: 0.61^b^ SE_M_: 1.50^a^ S_diff_: 2.12
	Spatial Span	Task: Visual pointing recall	Higher	Low internal consistency: 0.82^a^ Low test – retest: 0.65^b^ SE_M_: 1.27^a^ S_diff_: 1.80
	Auditory Immediate Index	Index: Measuring auditory immediate memory	Higher	Adequate internal consistency: 0.93^a^ Adequate test – retest: 0.84^b^ SE_M_: 3.97^a^ S_diff_: 5.61
	Auditory Delayed Index	Index: Measuring auditory delayed memory	Higher	Adequate internal consistency: 0.90^a^ Adequate test – retest: 0.82^b^ SE_M_: 4.74^a^ S_diff_: 6.70
	Visual Immediate Index	Index: Measuring visual immediate memory	Higher	Adequate internal consistency: 0.85^a^ Adequate test – retest: 0.74^b^ SE_M_: 5.81^a^ S_diff_: 8.22
	Visual Delayed Index	Index: Measuring visual delayed memory	Higher	Adequate internal consistency: 0.87^a^ Adequate test – retest: 0.74^b^ SE_M_: 5.41^a^ S_diff_: 7.65
	Immediate Memory index	Index: Measuring immediate memory	Higher	Adequate internal consistency: 0.92^a^ Adequate test – retest: 0.84^b^ SE_M_: 4.24^a^ S_diff_: 3.17
	Auditory Recognition Delayed Index	Index: Measuring recognition	Higher	Low internal consistency: 0.74^a^ Low test – retest: 0.60^b^ SE_M_: 7.65^a^ S_diff_: 10.82
	General Memory Index	Index: A global measure of delayed memory	Higher	Adequate internal consistency: 0.93^a^ Adequate test – retest: 0.87^b^ SE_M_: 3.97^a^ S_diff_: 5.61
	Working Memory Index	Index: Measuring working memory	Higher	Adequate internal consistency: 0.85^a^ Adequate test – retest: 0.70^b^ SE_M_: 5.81^a^ S_diff_: 8.22
Processing Speed Index (PSI) of the Wechsler Adult Intelligence Scale IV (WAIS-IV)^2^	Symbol Search	Task: Search for symbols in rows of symbols	Higher	Low internal consistency: 0.73^a^ Adequate test – retest: 0.75^c^ SE_M_: 1.56^a^ S_diff_: 2.21
	Symbol Search errors	–	Lower	–
	Coding	Task: Write correct symbols under numbers	Higher	Internal consistency: 0.84^a^ Adequate test – retest: 0.83^c^ SE_M_: 1.20^a^ S_diff_: 1.70
	Coding errors	–	Lower	–
	Processing Speed Index	Index: Measuring processing speed (and attention)	Higher	Adequate internal consistency: 0.87^a^ Adequate test – retest: 0.87^c^ SE_M_: 5.41^a^ S_diff_: 7.65
d2-R Test of Attention – Revised^3^	Processed Targets	Task: Search for d’s with marks in rows of letters with marks	Higher	–
	Errors	–	Lower	–
	Accuracy (% errors)	–	Lower	–
	Corrected total score	Measuring attention, (and processing speed and response inhibition)	Higher	High test – retest: 0.87 SE_M_: 40.1^d^ S_diff_: 56.71
	Concentration Performance	Measuring concentration	Higher	High test – retest: 0.91 SE_M_: 17.6^d^ S_diff_: 24.89
Delis-Kaplan Executive Function System (D-KEFS)^4^:				
Verbal Fluency Test	Condition 1: Phonemic/ Letter Fluency	Task: Words with specific first letter Measuring word fluency	Higher	High internal consistency: 0.90^e^ Moderate test – retest: 0.76^f^ SE_M_: 0.97^e^ S_diff_: 1.37
	Condition 2: Semantic/ Category Fluency	Task: Animals and boy’s names Measuring word fluency	Higher	Moderate internal consistency: 0.76^e^ Good test – retest: 0.81^f^ SE_M_: 1.48^e^ S_diff_: 2.09
	Condition 3: Category Switching	Task: Switch between furniture and fruits Measuring cognitive flexibility	Higher	Moderate internal consistency: 0.68^e^ Low test – retest: 0.49^f^ SE_M_: 1.71^e^ S_diff_: 2.42
	Repetition Errors	–	Lower	–
	Category Errors	–	Lower	–
Design Fluency Test	Condition 1: Filled Dot	Task: Draw different designs Measuring design fluency	Higher	Moderate test – retest: 0.62^f^ SE_M_: 1.94^g^ S_diff_: 2.74
	Condition 2: Empty Dots Only	Task: Draw different designs without the use of filled dots Measuring design fluency and response inhibition	Higher	Moderate test – retest: 0.73^f^ SE_M_: 1.98^g^ S_diff_: 2.80
	Condition 3: Switching	Task: Switch between filled and empty dots Measuring cognitive flexibility	Higher	Low test – retest: 0.22^f^ SE_M_: 2.47^g^ S_diff_: 3.49
	Repetition Errors	–	Lower	–
	Category Errors	–	Lower	–
Trail Making Test	Condition 1: Visual search	Task: Cross 3’s among numbers and letters Measuring attention, and processing speed	Lower	Low test – retest: 0.55^f^
	Condition 2: Numbers	Task: Draw line between numbers in order among numbers and letters Measuring attention and processing speed	Lower	Low test – retest: 0.54^f^
	Condition 3: Letters	Task: Draw line between letters in order among numbers and letters Measuring attention and processing speed	Lower	Low test – retest: 0.48^f^
	Condition 4: Number-Letter	Task: Switch between numbers and letters Measuring cognitive flexibility	Lower	Low test – retest: 0.36^f^
	Condition 4 Errors	–	Lower	–
	Condition 5: Motor Speed	–	Lower	Moderate test – retest: 0.73^f^
	Combined Number + Letter Sequencing	–	Lower	Moderate internal consistency: 0.78^e^ Moderate test – retest: 0.64^f^ SE_M_: 1.41^g^ S_diff_: 1.99
Wisconsin Card Sorting Test Revised and Expanded (WCST)^5^	Trials Administered	Task: Matching cards to key cards based on color, form, or number	Lower	–
	Correct trials	–	Higher	–
	Errors	–	Lower	Generalizability coefficient: 0.71 SE_M_: 8.08
	Perseverative Responses	Measuring cognitive inflexibility	Lower	Generalizability coefficient: 0.53 SE_M_: 10.28
	Perseverative Errors	Measuring cognitive inflexibility	Lower	Generalizability coefficient: 0.52 SE_M_: 10.39
	Non-perseverative Errors	–	Lower	Generalizability coefficient: 0.72 SE_M_: 7.94
	Conceptual Level Responses	–	Higher	–
	Categories Completed	–	Higher	–
	Failure to Maintain Set	–	Lower	–

^1^ Information from *WAIS-III WMS-III Technical Manual* (2002), re-test interval WMS-III = 35.6 days; ^2^ Information from *WAIS-IV Technical and Interpretive Manual* (2008), re-test interval WAIS-IV = 22 days; ^3^ Information from d2 Test of Attention in Danish *d2-testen – en vurdering af opmærksomhed of koncentration* (2006), re-test interval d2 test = 8–9 weeks; ^4^ Information from *D-KEFS Technical Manual* (2001), re-test interval D-KEFS = 25 days; ^5^ Information from *Wisconsin Card Sorting Test Manual – Revised and Expanded* (1993), re-test interval WCST = 32.61 days

*SE*_*M*_ Standard errors of measurement (reported in scaled-score units for subtests (for WMS-III, WAIS_IV, and D-KEFS), in index units for index scores (for WMS and WAIS), and in total score units (for d2 test and WCST))

*S*_*diff*_ Estimated standard error of the difference scores (calculated based on Iverson and Grant (2001))

^a^ Reported in relation to age 35–44 years; ^b^ Reported in relation to age 16–54 years; ^c^ Reported in relation to age 30–54 years; ^d^ Reported for whole sample 20–60 years; ^e^ Reported in relation to age 30–39 years; ^f^ Reported in relation to age 20–49 years; ^g^ Reported for whole sample 8–89 years

### Neuropsychological findings

Table 3 gives an overview of the timeline of the patient’s raw scores and scaled scores on the test battery. Table 4 presents the patient’s norm scores and percentiles on the WMS-III, the WAIS-IV PSI, and the d2-R. Table 5 presents the patient’s WCST scores at 2 weeks after admission (T_0_). Information on scoring are presented below each of the tables.

**Table 3:** The patient’s neuropsychological performance raw scores and scaled scores at two weeks after admission (T_0_), at re-test (T_1_), and at follow-up (T_2_)

Instrument	Raw scores			Scaled scores		
	T_**0**_	T_**1**_	T_**2**_	T_**0**_	T_**1**_	T_**2**_
**WMS-III**						
Logical Memory I	62	61	66	16	16	17
Logical Memory II	42	46	43	17	18	17
Verbal Paired Associated I	32	32	32	16	16	16
Verbal Paired Associated II	8	8	8	13	13	13
Faces I	42	41	45	12	11	15
Faces II	44	42	45	14	12	15
Family Pictures I	56	62	59	13	15	14
Family Pictures II	56	62	39	12	16	8
Letter-Number Sequencing	11	9	10	10	8	9
Spatial Span	18	13	16	11	8	10
**d2-R**						
Processed Targets	426	420	451			
Errors	3	0	4			
Accuracy (% errors)	0.70	0.00	0.89			
Corrected total score	423	420	447			
Concentration Performance	175	176	185			
**WAIS-IV Processing Speed**						
Symbol Search	28	28	35	7	8	10
Symbol Search Errors	3	0	1			
Coding	65	63	59	10	9	9
Coding Errors	0	0	1			
**D-KEFS**						
**Verbal Fluency Test:**						
Condition 1: Phonemic Fluency	49	60	56	13	16	15
Condition 2: Semantic Fluency	58	55	62	17	16	19
Condition 3: Category Switching	17	14	16	14	10	12
Repetition Errors	2	7	3	10	4	9
Category Errors	0	0	1	13	13	11
**Design Fluency Test**						
Condition 1: Filled Dot	10	14	15	10	13	14
Condition 2: Empty Dots Only	15	12	13	13	11	12
Condition 3: Switching	8	8	6	10	10	8
Repetition Errors	0	4	3	13	11	12
Category Errors	2	2	3	12	12	11
**Trail Making Test**						
Condition 1: Visual search	23	21	19	9	10	11
Condition 2: Numbers	55	46	41	3	6	7
Condition 3: Letters	32	30	36	10	10	9
Condition 4: Number-Letter	90	79	111	9	10	6
Condition 4 Errors	0	0	0			
Condition 5: Motor Speed	31	22	24	10	12	11

D-KEFS scaled scores (in relation to age and total sample): The mean is 10. The standard deviation is 3. 8 to 12 is considered average. 7 or lower is considered below average and 13 or higher above average

**Table 4:** The patient’s norm scores and percentiles on WMS-III, WAIS-IV Processing Speed Index (PSI), and d2-R at two weeks after admission (T_0_), at re-test (T_1_), and at follow-up (T_2_)

Instrument	Norm scores			Percentiles			95% confidence intervals		
	T_**0**_	T_**1**_	T_**2**_	T_**0**_	T_**1**_	T_**2**_	T_**0**_	T_**1**_	T_**2**_
**WMS-III Indexes:**									
Auditory Immediate	138	138	142	99	99	99.7	128–143	128–143	132–146
Auditory Delayed	132	108	132	98	70	98	119–137	98–116	119–137
Visual Immediate	115	118	127	84	88	96	102–123	105–125	112–132
Visual Delayed	118	125	109	88	95	73	105–125	111–131	97–118
Immediate Memory	132	134	142	98	99	99.7	121–137	123–139	131–146
Auditory Recognition Delayed	120	130	120	91	98	91	104–126	111–133	104–126
General Memory	130	125	125	98	95	95	119–135	115–131	115–131
Working Memory	102	88	96	55	21	39	92–111	80–99	98–106
**WAIS-IV PSI**	93	93	98	32	32	45	84–104	84–104	89–108
**d2-R**									
Processed Targets				21	18	34			
Errors				> 90	99	90			
Accuracy (% errors)				> 90	99	90			
Corrected total score				31	27	42			
Concentration Performance				42	42	54			

WMS-III norm scores (in relation to age): The mean is 100. 69 and below is extremely low. 70 to 79 is borderline. 80 to 89 is a low average. 90 to 109 is average. 110 to 119 is a high average. 120 to 129 is superior. 130 and above is very superior. WAIS-IV Processing Speed Index norm score (in relation to age): The mean is 100. 69 and below is extremely low. 70 to 79 is borderline. 80 to 89 is a low average. 90 to 109 is average. 110 to 119 is a high average. 120 to 129 is superior. 130 and above is very superior. d2 percentiles (in relation to age): The mean is 50

**Table 5:** The patient’s scores on the WCST at two weeks after admission T_0_

Wisconsin Card Sorting Test	Raw scores	Standard scores	Percentiles
Trials administered	128		
Correct	78		
Errors	50	79	8
Perseverative Responses	52	55	< 1
Perseverative Errors	36	65	1
Non-perseverative Errors	14	94	34
Conceptual level responses	61		
Categories Completed	1		< 1
Failure to Maintain Set	4		< 1

WCST standard scores (in relation to years of education): Standard scores greater than three standard deviations below the mean are considered in the severely impaired range (less than or equal to 54); 55 to 61 are in the moderately-to-severely impaired range; 62 to 69 are in the moderately impaired range; 70 to 76 are in the mildly-to-moderately impaired range; 77 to 84 are in the mildly impaired range; 85 to 91 are in the below average range; 92 to 106 are in the average range; standard scores equal to or greater than 107 are in the above-average range

*Note.* The standard score for non-perseverative errors was average since most of her errors were perseverative

### Memory performance on WMS-III

The patient’s scores on WMS-III indicate average to very superior auditory, visual, immediate and general memory performance (108 to 142; Mean: 100), and low average to average working memory (Table 4). The technical manual for WMS-III reports adequate test – retest reliability for all indexes in the age group 16–54 years, except for the Auditory Recognition Delayed Index ( [33]; Table 2). Estimated standard error of difference (S_Diff_) scores were calculated based on Iverson and Grant ( [34]; Table 2). Differences between the three assessments are outlined here. Her scores on the Auditory Delayed Index decreased more than S_diff_: 6.70 from 132 (very superior) at 2 weeks after admission (T_0_) to 108 (average) at re-test (T_1_) and increased again to 132 (very superior) at follow-up (T_2_). Her scores on the Visual Immediate Index increased slightly more than S_diff_: 6.70 from 118 (high average) at re-test (T_1_) to 127 (superior) at follow-up (T_2_). Her scores on the Visual Delayed Index decreased more than S_diff_: 7.65 from 125 (superior) at re-test (T_1_) to 109 (average) at follow-up (T_2_). Her scores on the Immediate Memory Index increased more than S_diff_: 3.17 from 134 (very superior) at re-test (T_1_) to 142 (very superior) at follow-up (T_2_). Her scores on the Working Memory Index decreased more than S_diff_: 8.22 from 102 (average) at 2 weeks after admission (T_0_) to 88 (low average) at re-test (T_1_). The scores on the rest of the indexes did not change more than the estimated S_diff_ scores between time points.

### Cognitive flexibility on D-KEFS and WCST

Overall, she performed above average on the Verbal Fluency Test (Table 3) at all three test times compared to the normative population for age, except for her performance at re-test (T_1_) on the switching condition, which was decreased more than S_diff_: 2.42 to average, and the high number of repetition errors (7; below average) at re-test (T_1_) and (3; average) at follow-up (T_2_).

She performed average to above average on the Design Fluency Test at all three test sessions (Table 3). However, the switching condition score was lower [6] at follow-up (T_2_) compared to 8 at 2 weeks after admission (T_0_) and re-test (T_1_), though still average.

During follow-up (T_2_) on the Trail Making Test (Table 3), her performance on the Number-Letter Sequencing test, measuring cognitive flexibility, was below average (111 s), in spite of being average at 2 weeks after admission (T_0_; 90 s) and re-test (T_1_; 79 s). The numbers condition was very low at T_0_ (55 s; below average), improving somewhat at re-test (T_1_; 46 s; below average) and follow-up (T_3_; 41 s; below average). We have no explanation for this result. On the other conditions, her performance was average at all three test times on the Trail Making Test.

Her scores on the WCST (Table 5) 2 weeks after admission (T_0_) place her in the mild to moderately-to-severely range of impairment on cognitive flexibility according to this task. She completed one out of six categories (< 1st percentile). She made 52 perseverative responses (< 1st percentile; standard score 55; moderately-to-severely impaired range). She committed 50 errors (8th percentile; standard score 79: mildly impaired range), of which 36 were perseverative errors (1st percentile; standard score 55: moderately impaired range).

### WAIS-IV processing speed

The scores on the Processing Speed Index (Table 4) were average compared to the normative population for age at all three test times. There were no relevant differences between time points. She scored 93 at admission (T_0_) and re-test (T_1_) and 98 at follow-up (T_2_).

### d2-R test of attention

At 2 weeks after admission (T_0_) and re-test (T_1_), she had a small number of processed targets (426 and 420), 18th to 21st percentile (Tables 3 and 4), her concentration performance was 175 and 176 corresponding to the 42nd percentile and she committed three and no errors respectively (> 90th percentile). At follow-up (T_2_), her concentration performance was above the mean (185; 54th percentile) but not increased more than S_diff_: 24.89. The total processed targets score was still low (451; 34th percentile), and she committed few errors (four; 90th percentile).

## Discussion and conclusions

The patient exhibited average to very superior performance on verbal fluency, design fluency, processing speed, and memory. However, her working memory performance was low average. Her attention and concentration performance were below average to average, and her performance on cognitive flexibility tasks were average to moderately-to-severely impaired.

The present case report demonstrates surprisingly good cognitive performance in a patient with severe and enduring AN with extremely low BMI varying between 7.7 and 9.3 during the study period of 1 year. However, some of her executive functions seem to be impaired. This is in line with previous research on patients with AN [5, 8]. The present results suggest that her working memory was normal (low average) in line with previous studies [35, 36]. However, her working memory performance was lower compared to the rest of her memory performance, which was average to very superior. The results from the D-KEFS indicate average to above-average performance with perhaps somewhat weaker cognitive flexibility (below average to average). On the other hand, the results from the WCST indicate impairment in cognitive flexibility. The overall differences in performance between the three assessments were minimal. This indicates that the minor differences in BMI between the test assessments did not significantly affect her cognitive performance, as expected.

### Impaired cognitive flexibility

It could be that impaired cognitive flexibility existed prior to the illness as a premorbid trait as suggested previously [10], or that the malnutrition has affected the patient’s cognitive flexibility. Since we are missing data on her premorbid level, we cannot draw any firm conclusions.

Impaired cognitive flexibility has previously been reported in patients with AN with higher BMI [37], indicating that impairments in cognitive flexibility do not necessarily relate to undernutrition. In patients with AN who had recovered from the illness, cognitive flexibility was in the normal range in this study. However, other studies found that individuals who recovered from AN exhibited more or less impaired executive functioning [10]. Longitudinal research on the relationship between different BMI states and cognitive performance is highly needed.

Impaired cognitive flexibility may also play a role in the perpetuation of AN. Impaired cognitive flexibility has been suggested as a maintenance factor [38] and a factor related to lack of illness insight characteristic of patients with restrictive AN [39]. Lack of illness insight could be related to treatment resistance [40]. The patient’s low scores on EDI-3 subscales also reflect a discrepancy between illness severity and self-reported symptoms. This discrepancy or ambivalence is part of the nature of the disorder reflected in the low motivation for recovery and high number of dropouts from treatment alongside an expressed desire to change [41].

### Cognitive adaptation in anorexia nervosa

Survival of long-term starvation is only possible due to extensive adaptive endocrine and metabolic alterations [42]. How these alterations affect cognitive functions still remains to be clarified. Well-designed longitudinal studies on severely underweight patients with a long illness duration are lacking. However, the present case report suggests that essential preservation of some cognitive functions occurs even in extreme chronic semi-starvation.

The mechanisms allowing for such preservation remains a subject of speculation. Links can be made to research on neuroplasticity and functional reorganization of cognitive functions after brain injury since patients with AN have white matter alterations [43]. Research shows that brain maturation processes of especially the prefrontal cortex continue until people are approximately 25 years old [44]. Nutritional status seems to impact this brain maturation [44]. Executive functions associated with the prefrontal cortex could therefore be affected by undernutrition during development of prefrontal connections in the brain in adolescence and young adulthood. Thus, impairment on executive functions may not arise until adulthood in patients with AN. This is in line with research that found no cognitive flexibility impairment in children and adolescents with AN but impairments in adults with AN [19, 20]. The literature indicates that other cognitive functions associated with the prefrontal cortex, such as memory, are also impaired in adults with AN [3]. However, overall, this literature is not as explicit as the literature showing cognitive flexibility impairment in adults with AN. The ambiguity in the literature indicates differences between cognitive functions related to the prefrontal cortex in patients with AN. It might be that some prefrontal connections potentially being affected during low weight in adolescence could be reorganized or “compensated for” with time as is possible with reorganization or apparent functional recovery after brain injury [45]. In that case, cognitive performance could be regained after impairment has occurred. Some dimensions of cognitive flexibility might, however, be more difficult to compensate for. This could explain specific cognitive flexibility impairment in patients recovered from AN [10] and explain that the patient in the present case report performed normal and superior on some functions associated with prefrontal connections (memory and verbal fluency) but poorer on cognitive flexibility. We therefore suggest that reorganization of some cognitive functions can occur in spite of persisting low weight in patients with AN. In line with the possibility of cognitive reorganization in AN, Cognitive Remediation Therapy seems to improve executive functioning in patients with AN [46]. The suggested theory of cognitive adaptation may therefore not be specific to persisting low weight in AN. However, fast, substantial weight-loss could affect cognitive performance differently than persisting low weight. Therefore, studies on starving healthy subjects, including the Minnesota Semi-Starvation Experiment [16], could show different results than studies on patients with severe and enduring AN. Likewise, studies on patients with short illness duration might find different results than studies of patients with enduring AN. It is also unclear if patients developing AN in adulthood will display the same cognitive impairments. In line with these reflections, a case report of a 27-year-old Japanese woman in a coma, with BMI of 8.5 kg/m^2^ at admission, describes a patient with AN where the outcome of severe malnutrition was persistent neurologic sequelae [47]. The woman developed AN at the age of 21 years where the patient in the present case report was diagnosed at the age of 10 years. The difference in age of onset, duration of illness, and/or manner of weight-loss (fast, substantial weight-loss compared to persisting low weight) may have resulted in different outcomes for the women. It is, however, also a possibility that the patient in the present case report might have an extreme phenotype which enables her to perform well in spite of her being extremely underweight.

We cannot say how high the patient’s scores on the neuropsychological test battery might be if she had not been as malnourished. We assume the patient would perform better on cognitive flexibility tasks, that her processing speed and working memory would be higher, and that she would be able to concentrate better had she not been malnourished. This is somewhat supported by previous research. Although the literature suggests impaired cognitive performance in patients with AN, the reported impairments were limited compared to healthy subjects [8, 48]. Furthermore, it may be that severely underweight patients with AN have a higher verbal IQ [49], which does not, however, exclude the possibility of specific cognitive impairments [50]. This could explain the patient’s high memory performance (and probably global IQ) alongside specific impairment in cognitive flexibility on the WCST. This case may therefore not differ from other patients with severe AN regarding cognitive performance. It may be that the superior performance related to some cognitive functions is a trait of severely underweight patients with AN and/or that a cognitive adaptation to enduring AN increases performance to the premorbid level. In this case, (regained) superior performance of some cognitive functions (i.e. memory and verbal IQ) can exist alongside cognitive impairment in others (i.e. cognitive flexibility). This may change our view of the cognitive profile and its development in patients with severe and enduring AN.

Regardless, the fact that we were able to test the patient in the present case, raises a discussion as to whether she and others with extremely low weight may be responsive to psychotherapy as well. In the present case, the patient underwent psychotherapy for several years albeit without any impact on her weight. More research focusing on the validation of neuropsychological tests including investigation of the practice effect in this patient population is needed.

The individual scores on neuropsychological tests should always be interpreted with care. Factors other than persisting low weight may affect neuropsychological performance (e.g. dehydration, stress, depression, and anxiety). In the present case, the patient did express depressive symptoms corresponding to moderate depression, which might have influenced results on impairment in cognitive flexibility. Furthermore, the patient might experience other issues related to cognitive performance in daily life, which cannot be discovered in a neuropsychological assessment context.

Obviously, conclusions can never be drawn from one case. However, since the neuropsychological testing included a broad range of tests and was repeated three times during a year, the present case report is valid as a basis for reflecting on the affected individual’s cognitive performance at this stage. The present case report demonstrates that cognitive functions may be largely preserved under extreme chronic malnutrition or that cognitive functioning may be regained (reorganized) in spite of extreme chronic malnutrition. More research on patients with AN with extremely low BMI (< 10) is needed to determine whether cognitive performance is affected by starvation and malnutrition.

## Data Availability

All data analyzed during this study are included in this published article in tables or text. Raw data in a fully anonymized version is available from the corresponding author on reasonable request.

## Abbreviations

AN: Anorexia nervosa
IQ: Intelligence quotient
BMI: Body mass index
BDI-II: The Beck Depression Inventory II
EDI-3: The Eating Disorder Inventory 3
WMS-III: The Wechsler Memory Scale III
WAIS-IV: The Wechsler Adult Intelligence Scale IV
PSI: The Processing Speed Index
D-KEFS: The Delis-Kaplan Executive Function System
WCST: The Wisconsin Card Sorting Test Revised and Expanded
